# Non-coding RNAs Regulate the Pathogenesis of Aortic Dissection

**DOI:** 10.3389/fcvm.2022.890607

**Published:** 2022-04-15

**Authors:** Yu-Yuan Hu, Xin-Meng Cheng, Nan Wu, Yang Tao, Xue-Ning Wang

**Affiliations:** Division of Cardiovascular Surgery, Third Hospital of Shanxi Medical University, Shanxi Bethune Hospital, Shanxi Academy of Medical Sciences, Tongji Shanxi Hospital, Taiyuan, China

**Keywords:** aortic dissection, non-coding RNAs, pathogenesis, therapeutic targets, ceRNA network

## Abstract

Aortic dissection (AD) is a fatal cardiovascular disease. It is caused by a rupture of the aortic intima or bleeding of the aortic wall that leads to the separation of different aortic wall layers. Patients with untreated AD have a mortality rate of 1–2% per hour after symptom onset. Therefore, effective biomarkers and therapeutic targets are needed to reduce AD-associated mortality. With the development of molecular technology, researchers have begun to explore the pathogenesis of AD at gene and protein levels, and have made some progress, but the pathogenesis of AD remains unclear. Non-coding RNAs, such as microRNAs, lncRNAs, and circRNAs, have been identified as basic regulators of gene expression and are found to play a key role in the pathogenesis of AD. Thus, providing a theoretical basis for developing these non-coding RNAs as clinical biomarkers and new therapeutic targets for AD in the future. Previous studies on the pathogenesis of AD focused on miRNAs, but recently, there have been an increasing number of studies that explore the role of lncRNAs, and circRNAs in AD. This review summarizes the existing knowledge on the roles of various non-coding RNAs in the pathogenesis of AD, discusses their potential role as clinical biomarkers and therapeutic targets, states the limitations of existing evidence, and recommends future avenues of research on the pathogenesis of AD.

## Introduction

Aortic dissection (AD) is a life-threatening condition in which the lining of the aorta is torn, causing the parietal layer of the aorta to separate (1). Anatomically, AD is classified depending on the position of the rupture (2). According to the Stanford classification, type A aortic dissection (AAD) disease originates in the ascending aorta and type B AD disease originates in the descending aorta (3). AD is associated with high mortality and morbidity (4). The current incidence of AD is approximately three cases per 100,000 people per year. Approximately 75% of patients with acute type A aortic dissection (TAAD) die within 2 weeks without timely treatment (5, 6). Thus, emergency thoracotomy is currently the best solution for saving the lives of patients with TAAD (7). However, patients may suffer many complications during open surgical treatment (8). Hence, there is an urgent need to further explore the key molecular mechanisms of AD to identify effective therapeutic targets (7).

Although research on AD is ongoing for decades, limited knowledge is available about the factors affecting its progress and regulatory mechanisms at the gene level. Several reported factors can lead to the occurrence and development of AD. These factors are broadly grouped into two categories, namely genetic or cytological. Genetic diseases affecting the aortic wall structure primarily include Marfan syndrome, Ehlers–Danlos syndrome, and familial thoracic aortic aneurysm (9). Regarding cytological factors, recent studies show that the pathological mechanism of AD mainly includes aortic medial cystic necrosis and degenerative changes (10). Which may be related to the changes in proliferation, migration, or apoptosis of aortic vascular smooth muscle cells and changes in extracellular matrix components (11). Multiple studies have shown that VSMCs not only produce extracellular matrix (ECM), but also participate in the release and maturation of matrix metalloproteinases (MMPs) and inhibitors of matrix metalloproteinases (TIMPs), which in turn control the integrity and degradation of the extracellular fibrous structure of the aortic wall (12). However, the transition from a contractile phenotype to a synthetic phenotype increases the proliferation and migration of cells to compensate for vascular smooth muscle cells (VSMCs) loss in AD (13). VSMC apoptosis and ECM destruction in the aortic wall are accompanied by an increased degree of inflammation (14). In general, the progressions in endothelial cells (ECs), VSMCs, and inflammatory cells are firmly connected with AD improvement (15). There is no absolute single cause for the occurrence of AD, rather AD may result from a combined effect of environmental and genetic factors (16). At present, the following genes are identified as pathogenic for AD: TGFBR2, TGFBR1, MYH11, ACTA2, MYLK, SMAD3, and so on (17).

Recently, an increasing number of studies have focused on the involvement of non-coding RNAs (ncRNAs) in various diseases, such as tumors and cardiovascular diseases (18, 19). ncRNAs are divided into two subclasses according to length: small or short ncRNAs (sncRNAs, 18–200 nt) and long ncRNAs (lncRNA; > 200 nt) (20). sncRNAs mainly include microRNAs (miRNAs), piwi—interaction RNAs (piRNAs), small interfering RNAs (siRNAs), and circular RNAs (circRNAs). Studies have found that ncRNA is a key regulator of gene expression under physiological and pathological conditions and interacts with DNA, RNA, and proteins (21). In molecular biology, miRNAs are involved in the proliferation, apoptosis, migration, and phenotypic transition of VSMCs (22). Therefore, an increasing number of studies on the pathogenesis of AD have begun to focus on miRNAs. In the pathophysiology of blood arteries, studies have demonstrated that lncRNAs can modulate the expression level and biological roles of miRNAs as endogenous sponges (23). Studies have also found that circRNAs can directly regulate transcription by interacting with mRNAs or lncRNAs, sponging mRNAs, or RNA binding proteins (RBPs) (24, 25). Therefore, researchers speculate that the circRNA-miRNA-mRNA pathway may play an important role in cardiovascular diseases. In this review, we summarized the various ncRNAs that are identified to be involved in the development of AD, discussed the significance and limitations of each ncRNA in AD research, and stated recommendations and prospects for future studies. In addition, the potential role of miRNAs, lncRNAs, and circRNAs as clinical biomarkers and therapeutic agents is also discussed.

## MicroRNAs in Aortic Dissection

miRNAs are endogenous ncRNAs with a length of approximately 20–25 nucleotides. They play an important role in regulating cell differentiation, proliferation, migration, apoptosis, and other pathophysiological functions (26). Mature miRNAs usually regulate target mRNAs by combining with a short sequence located in the 3′UTR region of RNA, inhibiting the translation of target mRNAs according to the degree of complementarity, and guiding the degradation of target mRNAs (27). miRNAs have been revealed to play a key part in all biological processes, and aberrant miRNA expression has been linked to a variety of diseases, including neurodegenerative diseases, metabolic disorders, cancer, and cardiovascular diseases (28, 29). miRNAs are also involved in the proliferation, apoptosis, migration, and phenotype conversion of VSMCs (22). For example, researchers found that miRNA-22 inhibits vascular smooth muscle cell apoptosis in AD vascular remodeling by targeting p38 mitogen-activated protein kinase α (p38MAPKα) (30). Xue et al. found that miR-146a-5p stimulates VSMCs proliferation and migration by targeting SMAD4 (31). miRNAs are also involved in oxidative stress and inflammation. For example, Dai et al. found that miRNA-137 reduces the progression of spinal cord inflammation and oxidative stress by degrading neuronal differentiation 4 (NEUROD4) (32). In this review, we summarized a variety of miRNAs that can regulate the pathogenesis of AD, explored their mechanisms and limitations in the pathogenesis of AD, and provided references and ideas for future research (Table 1).

**TABLE 1:** Selected ncRNAs implicated in aortic dissection.

ncRNAs	Cell type	Animal model	Upregulated or Downregulated	Target gene	Associated functions	References
miR-26	VSMC	NO	DOWN	HMGA2	Promote VSMC proliferation	(26)
miR-145	VSMC	NO	DOWN	CTGF	Promote VSMC proliferation	(7, 29)
miR-30a	VSMC	YES	UP	LOX	Reduced elastin	(33)
miR-134-5p	AoSMC	YES	DOWN	STAT5B ITGB1	Inhibit AoSMC migration	(36)
miR-107	AoSMC	NO	UP	ITM2C	Promote the proliferation of AoSMC	(41)
miR-146-5P	VSMC	NO	UP	SMAD4	Promote VSMC proliferation and migration	(21)
miR-22	VSMC	NO	DOWN	p38MAPKα	Inhibit VSMC apoptosis	(47)
miR-27a	EC	YES	DOWN	FADD	Inhibit EC apoptosis	(50)
LncRNA PVT1	HASMCs	YES	UP	miR-27b-3p	Promote VSMC apoptosis and phenotypic transition	(64)
CDKN2B-AS1	VSMC	NO	UP	miR320d STAT3	Inhibit VSMC proliferation	(70)
Lnc-OIP5-AS1	HAECs HASMCs	YES	UP	miR-143-3p/TUB	Inhibit the proliferation and migration of EC and VSMC	(56)
LncRNA H19	HASMCs	YES	UP	miR-193b-3p	Inhibit VSMC proliferation and promote apoptosis	(84)
Linc01278	VSMC	NO	DOWN	miR-500b-5p/ACTG2	Phenotype transformation, proliferation and migration	(89)
circTGFBR2	VSMC	YES	DOWN	miR29a /KLF4	Promote the proliferation and migration of VSMC	(99)
circMARK3	HASMCs	NO	UP	miR1273 /Fgr	Waiting for research	(101)
circRNA101238	SERUM	NO	UP	miR320a MMP9	Waiting for research	(103)

### MicroRNA-26b

**FIGURE 1:**
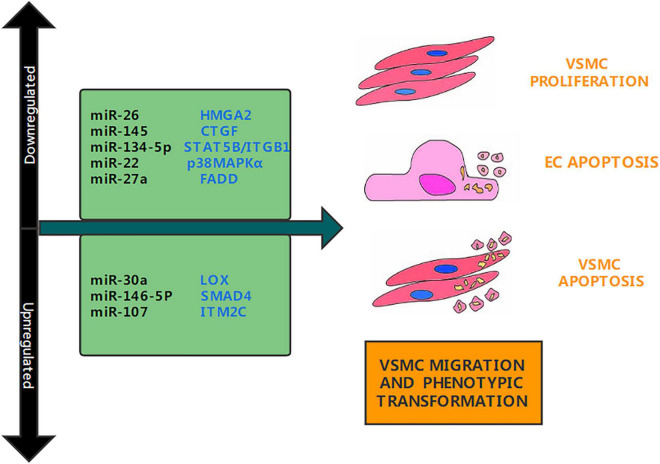
Aortic dissection associated miRNAs. Several studies have determined that miRNAs are upregulated or downregulated in aortic dissection. This figure shows the miRNAs involved in the pathogenesis of aortic dissection and their target genes, as well as their functions.

MicroRNA-26b (miR-26b) is a miRNA regulated by hypoxia, which is downregulated under hypoxic exposure (33). MiR-26b is a protective gene in cardiovascular diseases. Its overexpression can reduce myocardial hypertrophy. It can also reduce inflammation and myocardial remodeling in mice with myocardial infarction by inhibiting the MAPK pathway in combination with PTGS2 (34). Recently, Yang et al. found that miR-26b also plays an important role in AD. They divided TAAD patients into three groups: light-risk, intermediate-risk, and severe-risk, and discovered that miR-26b was reduced in the aortic wall tissue of TAAD patients, and its expression level was significantly lower in the intermediate-risk and severe-risk groups than in the light-risk group (Figure 1). Multiple *in vitro* experiments confirmed that miR-26b targeting high mobility group box 2 (HMGA2) promoted VSMCs proliferation and inhibited VSMCs apoptosis (35). These experiments tested whether the TGF/Smad3 signaling pathway is involved in the development of acute TAAD that is regulated by miR-26b, and discovered that miR-26b regulates the HMGA2 and TGF/Smad3 signaling pathways (36). However, there are still many issues worth investigating. An animal model has not been constructed to demonstrate the effect of miR-26b on the formation of aortic dissection in mice and the results of *in vitro* studies. It is also unclear whether miR-26b targets other genes. Future studies should explore the impact of miR-26b in animal experiments and on other target genes to identify its role in the regulation of other signaling pathways and development of TAAD.

### MicroRNA-145

miRNA-145 is a tumor suppressor miRNA, and research has found that it can target various anti-apoptotic molecules to participate in the regulation of the tumor cell apoptosis network (37). Recently, Li et al. found that miR-145 protects cardiomyocytes from hydrogen peroxide (H_2_O_2_)—induced apoptosis by targeting mitochondrial apoptosis (38). This has led researchers to focus on the role of miR-145 in cardiovascular disease. In a recent article, Li et al. found that miR-145 plays a key role in the pathogenesis of AD; They discovered that miR-145 is downregulated in TAAD and that overexpression of miR-145, through targeting connective tissue growth factor (CTGF), enhances the progression of TAAD (Figure 1). CTGF was discovered to inhibit the progression of TAAD by reversing the action of miR-145 (39). This is consistent with the study by Huang et al. They confirmed that miR-145 can induce VSMCs proliferation, migration and apoptosis by targeting Smad3 and participate in the pathogenesis of AD (11). Their research adds to our understanding of the molecular underpinnings of AD and looks at the prospect of using miRNAs as a preventative strategy for the disease.

### MicroRNA-30a

Lysyl oxidase (LOX) is a copper-dependent oxidative deaminase that can cross-link collagen and elastin (40). Studies have shown that LOX has an important function in the cardiovascular system and that LOX inactivation can cause aortic aneurysms in mice (41). Recently, Yu et al. found that the miR-30a-LOX axis plays an important role in the pathogenesis of AD (42). They confirmed that the expression of the miR-30a gene was increased in aortic specimens of patients with AD, and the expression of LOX and elastin protein was decreased. miR-30a can drastically lower the protein abundance of LOX and elastin in cultured VSMCs, according to several *in vitro* tests (Figure 1). These studies created a mouse model of AD and found that transfection with agomiR-30a enhanced the gene expression of miR-30a and inhibited the protein abundance of LOX and elastin in rat aorta. This verified the results of the *in vitro* experiments. In general, miR-30a overexpression enhanced the development of Ang II-induced AD in rats by targeting LOX directly. Therefore, miR-30a may be a drug target for the prevention of AD (43). However, it remains unknown whether miR-30a plays other roles in AD. Most importantly, miR-30a is known to inhibit the occurrence and development of various cancers. Inhibition of miR-30a to prevent AD may lead to an increase in the incidence of malignant tumors (42). Future research should look at whether controlling miR-30a in aortic diseases causes the occurrence of other diseases or has other impacts.

### MicroRNA-134-5p

miRNAs are endogenous non-coding RNAs that can target mRNAs, inhibit their translation or promote their degradation, and regulate gene expression at the post-transcriptional level (44). Growing proof suggests that miR-134-5p is dysregulated in a range of cardiovascular diseases. For example, Lu et al. found that miR-134-5p can target X-linked apoptotic protein inhibitor (XIAP) to regulate hypoxia/oxidative stress and apoptosis of cardiomyocytes under reperfusion injury (45). Recently, Wang et al. found that miR-134-5p is also involved in the pathogenesis of AD. They determined that the expression of miR-134-5p was extensively decreased in the aortic tissue of TAD sufferers and carried out *in vitro* experiments to verify that miR-134-5p overexpression can inhibit the migration of aortic smooth muscle cells (AoSMCs) (Figure 1). Thereafter, they constructed an miRNA-mRNA network to predict target genes and identified and verified STAT5B and ITGB1 as the downstream target genes (46). STAT5B is a member of the STAT family and is involved in regulating cell proliferation and differentiation (47). Studies have found that ITGB1 is involved in cell migration and invasion (48). Through cell function experiments, researchers found that miR-134-5p can target STAT5B and ITGB1 to promote VSMCs phenotype conversion, proliferation, and migration (46). Animal models have been additionally established to affirm the outcomes of the *in vitro* experiments. While this review is the first to explore the regulatory role of miR-134-5p in AD, future studies should not limit the investigation to the ITGB1 pathway and should examine other therapeutic targets in the STAT5B pathway and miR-134-5p.

### MicroRNA-107

A giant variety of research have determined that miRNA-107 plays multiple roles, including cell division, metabolism, and stress response, in different tissues and cells (49). For example, Jiang et al. found that miR-107 overexpression promoted the viability, migration, and proliferation of human osteosarcoma cells (50). Jiang et al. also found that miR-107 can target KRT1 to inhibit coronary atherosclerotic endothelial cell inflammation and endoplasmic reticulum stress through the Notch signaling pathway (50). Recently, Wang et al. discovered that miR-107-5p is significantly upregulated in the aortic tissues of AD patients, and revealed through bioinformatics analysis that integral membrane protein (ITM2C) may also be the goal of miR-107-5p. These results were confirmed by qRT-PCR and a collection of *in vitro* experiments that confirmed that overexpression of miR-107-5p promotes cell proliferation and inhibits cell apoptosis in human aortic smooth muscle cells (Figure 1). ITM2C was also discovered to be under-expressed in AD patients, and luciferase reporter analysis verified that ITM2C is the goal of miR-107-5p (51). However, no animal model of AD has been set up to affirm the effects of *in vitro* experiments. In addition, the effect of miR-107 in AD has not been discussed in detail, and only the aspect of apoptosis has been explained. Further research is needed to confirm whether miR-107 affects the migration of human aortic smooth muscle cells, phenotypic conversion, inflammatory response, and oxidative stress.

### MiR-146a-5p

Previous studies have shown that the downregulation of some goal genes (such as IRAK1, TRAF6, and SMAD4) mediated by miR-146a-5p helps tumorigenesis in some instances and inhibits tumorigenesis in different cases (52). Studies have also found miR146a-5p is involved in cell proliferation as well as migration, and therefore, may be related to the pathogenesis of AD (53). Recently, Xue et al. found that miR146a-5p plays an important role in AD. They observed that the expression of miR146a-5p used to be accelerated in the aortic tissue and circulation of sufferers with AD. A massive range of experiments established that overexpression of miR146a-5p will increase the proliferation and migration of VSMCs, while low expression reduces the migration and proliferation of VSMCs (Figure 1). Xue et al. also found that SMAD4 is a new goal of miR146a-5p in VSMCs, and verified that extraordinary expression of miR146a-5p can alternate the expression of SMAD4 in VSMCs (31). SMAD4 or DPC4 are tumor suppressor genes and key intracellular transcription mediators of transforming growth factor-β (TGF-β) (54); they can modify many key cell processes, for instance, migration, adhesion, cellular division, variation, tissue homeostasis, and embryogenesis (55). In general, miR146a-5p is involved in the nosogenesis of AD by way of directly targeting SMAD4 (31). While this review discusses few molecular mechanisms that may cause the pathogenesis of AD, several other mechanisms need to be investigated.

### MiR-22

MiR-22 has previously been confirmed as a tumor suppressor and is downregulated in a range of cancers (56). Bioinformatics research has shown that miR-22 restrain cell growth, migration as well as invasion (57). Recent research have additionally located that miR-22 performs a function in regulating multiple cellular processes in a variety of cardiovascular diseases. Xiao et al. found that miR-22 plays an important role in the pathogenesis of AD; they confirmed through qRT-PCR that miR-22 was appreciably downregulated in AD tissues and that downregulated of miR-22 extended apoptosis of VSMCs. Bioinformatic analysis revealed that p38MAPKα was once the goal of miR-22. Previous research have proven that oxidative stress is activated through the MAPK pathway, which affects the development of AD (58). To explore whether miR-22 and p38MAPKα have an effect on VSMC apoptosis, researchers transfected mimics, inhibitors, or small interfering RNA (siRNA) plasmids into VSMCs (Figure 1). The results showed that overexpression of p38MAPKα significantly increased apoptosis in human aortic smooth muscle cells (HASMCs). When miR-22 is downregulated, the promotion of apoptosis in HASMCs can be reversed by inhibiting the expression of p38MAPKα (30). However, that study had some limitations. First, the researchers did not find other potential targets of miR-22, and second, they did not explore in detail how p38MAPKα regulates VSMCs apoptosis. Future research should address these limitations.

### MiR-27a

The MiR-27 family consists of miR-27a and miR-27b, which are transcribed from different chromosomes with specific 3′ nucleotides. Studies have discovered that miR-27a, one of the two participants of the miR-27 family, is concerned in a variety of biological processes, such as cellular proliferation, apoptosis, invasion, migration, angiogenesis, and tumorigenesis (59). It has been established that miR-27a is a rich miRNA in endothelial cells and can regulate many functions of endothelial cells (60). Endothelial cells are largely unexplored in the study of AD pathogenesis; however, Sun et al. recently discovered that miR-27a can promote the development of AD by targeting endothelial cell apoptosis (Figure 1). Through fluorescence *in situ* hybridization, they found that miR-27a was mainly expressed in the inner membrane layer by and was significantly downregulated among patients with AD. A series of *in vitro* experiments confirmed that miR-27a inhibition promoted endothelial cell apoptosis, and miR-27a overexpression reduced endothelial cell apoptosis (61). Researchers performed protein chip analysis and KEGG pathway enrichment analysis and found that Fas-related death domain protein (FADD) is key to the apoptosis pathway. Previous studies have shown that the interaction between FADD and the death domain of Fas can activate apoptosis (62). According to the luciferase reporter analysis, FADD may be a downstream target of miR-27a. Researchers conducted a large number of *in vitro* experiments and established a mouse model, confirming that the knockdown of miR-27a can induce increased apoptosis of FADD and ECs, thereby increasing the incidence of AD (61). That study is of great significance, as it is the first to verify that AD is related to endothelial cell dysfunction and provides a deeper understanding of the pathogenesis of AD. However, it does not investigate whether miR-27a has other targets that regulate the occurrence of AD. miR-27a regulate FADD mechanism has not been a detailed exploration. Future studies must explore other targets of miR-27a and examine whether miR-27a can affect the occurrence and development of AD through oxidative stress or inflammation.

## LncRNAs in Aortic Dissection

Long non-coding RNAs (lncRNAs) are RNA transcripts that are longer than 200 nucleotides and do not have the ability to encode proteins (63, 64). Studies have observed that lncRNA can alter the expression degree and biological function of miRNA as an endogenous sponge in the pathophysiology of blood vessels (23). Further, lncRNAs can competitively adsorb some miRNAs as natural sponges, decreasing the binding of these miRNAs to goal genes and leading to adjustments in the expression ranges of these miRNA target genes (65, 66). For example, Wang et al. determined that lncRNA OIP5-AS1 can upregulate TUB through sponging miR-143-3p, which aggravates the damage of the aortic intima, media, and adventitia in the course of the incidence and development of AD (67). As new epigenetic regulatory molecules, lncRNAs have acquired large interest in the development of aortic diseases (68). In this review, we summarize a variety of lncRNAs that can regulate the pathogenesis of AD and discuss how they regulate the occurrence and development of AD. We also discuss the limitations of existing research. Finally, prospects for future research are proposed.

### LncRNA PVT1

**FIGURE 2:**
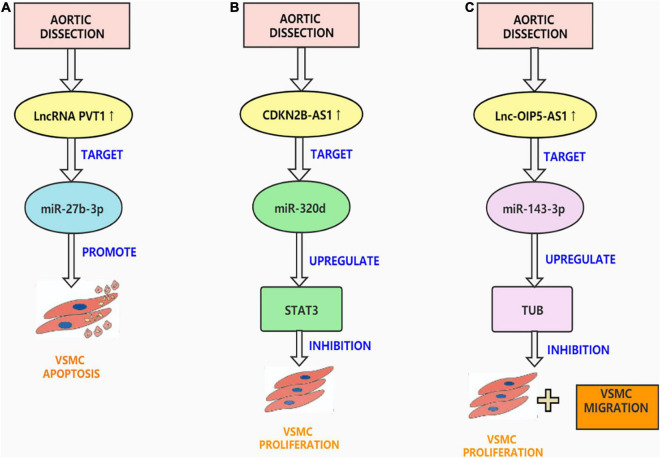
Aortic dissection associated lncRNAs. **(A)** LncRNA PVT1 is up-regulated in AD tissues, targeting miR-27b-3p promotes VSMC apoptosis, inhibits VSMC viability, migration and phenotypic transformation. **(B)** CDKN2BAS1 enables STAT3 expression in VSMCs by competitively sponging miR-320d and thus controlling the multiplication and apoptosis of VSMCs. **(C)** OIP5-AS1 can bind to Mir-143-3p as a ceRNA and inhibit the proliferation and migration of HAECs and HASMCs.

lncRNA plasmacytoma variant translocation 1 (PVT1) is placed on the sense chain of chromosome 51 8q24 and is an intergenic lncRNA (69). Previous studies have found that PVT1 is dysregulated in many diseases, such as cancer and cardiovascular diseases (70). PVT1 can act as a carcinogenic molecule in tumors. For example, Zhao et al. proposed that PVT1 can promote the hyperpiasia and migration of pancreatic cancer cells via sponging miR-448 (71). In cardiovascular diseases, PVT1 has been reported to modify the increase and migration of vascular endothelial cells (72). For example, Du et al. proposed that silencing PVT1 can inhibit the development of atherosclerosis through the MAPK/NF-κB pathway (73). This is consistent with the results of Guo et al. who showed that PVT1 knockdown can reduce vascular endothelial damage and atherosclerosis through the ERK/p38 pathway (74). Recently, Li et al. determined that PVT1 additionally performs an important position in AD. They found that miR-27b-3p is the binding site of PVT1 and verified it through luciferase reporter analysis. To examine the effect of PVT1 on the activity, migration, and phenotypic transition of HASMCs, they conducted a series of *in vitro* experiments. The outcomes confirmed that downregulation of PVT1 inhibited the viability, migration, and phenotype switching of PDGF-BB-treated HASMCs by targeting miR-27b-3p (75). They also established an AD animal mannequin to affirm the effects of *in vitro* experiments. Their research revealed the molecular mechanism of PVT1 in AD for the first time. However, the underlying mechanism is not yet fully understood. In the future, studies must explore whether there are other targets of PVT1 that affect the occurrence of AD (Figure 2A).

### CDKN2B-AS1

The lncRNA cyclin-dependent kinase inhibitor 2 B antisense RNA 1 (CDKN2B-AS1), also called ANRIL, is mainly expressed in tissues related to coronary heart disease, including the heart, vascular endothelial cells, and human monocyte-derived giant phages (76). CDKN2B-AS1 can regulate inflammation and the cell cycle and is considered to be the main susceptibility site for cardiovascular disease (77). Ma and Dong pointed out that CDKN2B-AS1 can sponge miR-143-3p to promote the proliferation and migration of human carotid smooth muscle cells (78). Additionally, Li et al. found that CDKN2B-AS1 inhibits metalloproteinase 10 (ADAM10) transcription through DNA methyltransferase 1 (DNMT1)-mediated ADAM10 DNA methylation, thereby preventing atherosclerotic inflammation (79). This is consistent with the study by Li et al. They confirmed the ceRNA network of CDKN2B-AS1 in coronary heart disease and found that CDKN2B-AS1 upregulates protein tyrosine phosphatase non-receptor type 7 (PTPN7) by absorbing miR-126-5p and inhibits the PI3K-Akt pathway, thus hindering the proliferation of and accelerating apoptosis of VSMCs induced by oxidized low-density lipoprotein (ox-LDL) (80). Notably, Zhao et al. found that CDKN2B-AS1 plays a regulatory role in the pathogenesis of AD. They found that the CDKN2B-AS1 was overexpressed in AD tissues, and several *in vitro* experiments confirmed that CDKN2B-AS1 overexpression can inhibit VSMCs proliferation. They also confirmed the ceRNA network of CDKN2B-AS1 in AD, and that CDKN2BAS1 enables STAT3 expression in VSMCs by competitively sponging miR-320d and thus controlling the multiplication and apoptosis of VSMCs (81). However, their study did not establish an AD animal model to verify the results of the *in vitro* experiments. It is also necessary to determine whether CDKN2BAS1 has other targets that can regulate the occurrence of AD (Figure 2B).

### Lnc-OIP5-AS1

OIP5 antisense transcript 1 (OIP5-AS1) is an lncRNA that is highly expressed in the nervous system (82). OIP5-AS1 partakes in the regulation of cell cycle transitions at different points and can act as a competitive endogenous RNA for a variety of miRNAs (83). Previous studies have shown that OIP5-AS1 is the causative gene in a variety of cancers, such as cervical cancer (84), lung cancer (85), and breast cancer (86). OIP5-AS1 is also involved in regulating cardiovascular disease; Zheng et al. found that OIP5-AS1 regulates atherosclerosis by inducing endothelial cell harm through the mir-98-5p/HNGB1 axis (87). Recently, Wang et al. found that OIP5-AS1 plays a significant role in AD. In AD and normal aortic tissue microarray analysis, OIP5-AS1 and miR-143-3p were selected for the study to predict and confirm TUB as a goal gene of miR-143-3p. Several *in vitro* experiments confirm that OIP5-AS1 can bind to miR-143-3p as competitive endogenous RNAs (ceRNAs) and inhibit the proliferation and migration of human aortic endothelial cells (HAECs) and HASMCs. Researchers established an animal model of AD to confirm the results of *in vitro* experiments. In general, OIP5-AS1 upregulates TUB via the sponge protein Mir-143-3p, exacerbating damage to the intima, media, and adventitia of the aorta during AD development (67). Their study is of great significance because it is the first to explore the regulatory role of lncRNAs in AD in the entire thickness of the aorta (intima, media, and adventitia). However, their clinical specimens were not sufficient, and there may be errors. In addition, it remains unknown whether OIP5-AS1 can sponge other miRNAs to regulate the occurrence and development of AD (Figure 2C).

### LncRNA H19

**FIGURE 3:**
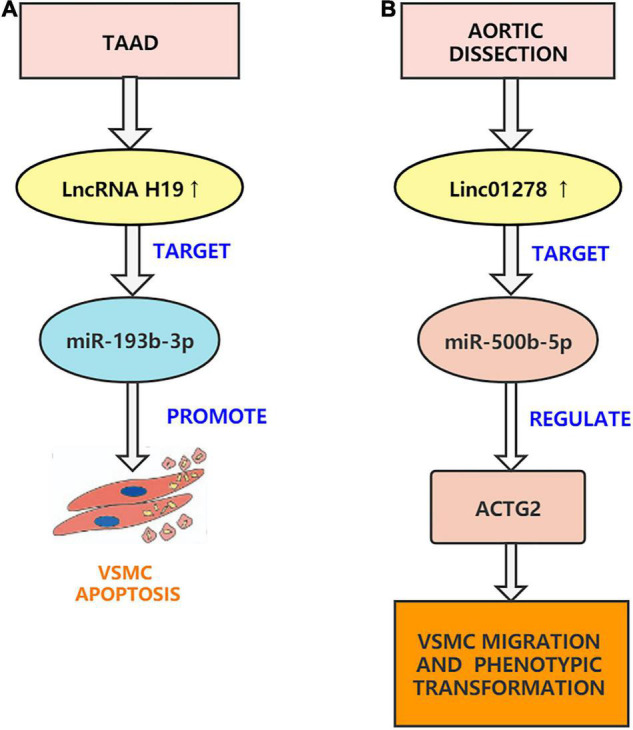
Aortic dissection associated lncRNAs. **(A)** H19 can regulate the function of VSMCs through sponging miR-193b-3p and can participate in the development of AD. **(B)** Linc01278 sponge miR-500b-5p can regulate ACTG2 and control VSMC phenotypic transition, proliferation, and migration.

Several studies have shown that the pathogenesis of AD is mainly related to VSMCs apoptosis, inflammation, and oxidative stress. LncRNA H19 is a ncRNA transcript of the H19 gene, which is proven to be a developmentally regulated RNA (88). Many research have additionally observed that lncRNA H19 performs an vital position in promoting vascular inflammation and promoting VSMCs apoptosis and migration. The expression of lncRNA H19 is only retained in the heart and bones, and its re-expression in different tissues is carefully associated to inflammatory diseases (89). Pan. found that increased expression of lncRNA H19 quickens atherosclerosis by activating inflammatory pathways, while H19 silencing inhibits ox-LDL treatment-induced *in vitro* adipogenesis and inflammation (90). Subsequently, Zhang et al. conducted an in-depth study and found that H19 knockdown inhibits proliferation and induces apoptosis by regulating miR-148b/WNT/β-catenin in vascular smooth muscle cells stimulated by ox-LDL (91). The two studies showed consistent results. In addition, Sun et al. found that downregulation of lncRNA H19 reduces atherosclerosis by inducing apoptosis of vascular smooth muscle cells (92). It is worth noting that researchers previously found that lncRNA H19 can promote the occurrence and development of abdominal aortic aneurysms by inducing VSMCs apoptosis (93). This is consistent with the study by Sun et al. who found that the overexpression of lncRNA H19 upregulated the pro-inflammatory factors IL-6 and MCP-1 to exacerbate aortic inflammation and promote the occurrence of aortic aneurysms (94). Recently, Ren et al. found that lncRNA H19 is concerned in the development of AD and that lncRNA H19 was once extensively upregulated in the thoracic aorta tissue of AD patients. Through a series of *in vitro* experiments, lncRNA H19 silencing was found to reduce the phenotypic transition and migration of HASMCs that were induced by platelet-derived growth factor BB (PDGF-BB). Ren et al. used the starBase database to predict that H19 might target miR-193b-3p and found that the downregulation of miR-193b-3p reversed the impact of shH19 on HASMC phenotype transition and migration. They additionally established an AD animal model to confirm the outcomes of *in vitro* experiments. In general, H19 can regulate the function of VSMCs through sponging miR-193b-3p and can participate in the occurrence of AD (95). Recently, Fan et al. also confirmed that H19 can inhibit proliferation and accelerate VSMCs apoptosis and ECM degradation (96). Based on previous studies, we can speculate that H19 may sponge other miRNAs to affect the development of AD. In short, through the efforts of researchers, H19 has become a molecular target for the treatment of AD (Figure 3A).

### Linc01278

Previous research has determined that linc01278 is concerned with the physiological and pathological processes of many cancers. For example, Xi et al. pointed out that linc01278 accelerates the progression of colorectal cancer through the miR-134-5p/KDM2A axis (97). Lin et al. pointed out that Linc01278 inhibits the development of papillary thyroid carcinoma by regulating the miR-376c-3p/DNM3 axis (98). Studies have also shown that lncRNAs can regulate a range of cell functions, which includes cell proliferation, apoptosis, migration, and VSMCs phenotypic transition, by sponging miRNAs (99). Additionally, Wang et al. found that linc01278 plays a role in AD and confirmed that the linc01278/miR-500b-5p/ACTG2 axis is the most relevant target gene through advanced bioinformatics analysis such as GEO2R analysis, gene ontology (GO), pathway enrichment analysis, and protein-protein interaction (PPI) network (100). Wang et al. found that the levels of linc01278 and ACTG2 in AD tissues decreased, and the expression of miR-500b-5p increased. After a series of *in vitro* experiments, it was confirmed that Si-linc01278 transfection promoted the migration and muscle growth of human aortic vascular smooth muscle cells, but this effect was reversed by the miR-500B-5P inhibitor. In addition, Si-linc01278 promoted the proliferation of VSMCs. Overexpression of linc01278 showed the opposite phenomenon. Therefore, Wang et al. inferred that the linc01278 sponge miR-500b-5p can regulate ACTG2 and control VSMCs phenotypic transition, proliferation, and migration (100). However, their study has limitations. First, animal models were not established for validation. Second, their study did not explain how miR-500b-5p regulates ACTG2 expression. Finally, they did not explore the effect of linc01278 on endothelial cells and outer membranes. Future research should address these limitations (Figure 3B).

## CircRNAs and Aortic Dissection

Circular RNA (circRNA) is a new kind of endogenous non-coding RNA. They are produced by the combination of the 3′ end and 5′ end and have a covalent closed loop structure, thus they are highly stable *in vivo* (101). Studies have found that circRNAs can interact with miRNAs to regulate gene expression after transcription or at the transcriptional level (102). Thereby, regulating various biological processes, such as cell proliferation, apoptosis, and migration (103). CircRNAs have been shown to act as sponges for miRNAs and may also potentially sponge RNA binding proteins (RBPs) (104). In addition, an increasing number of studies have shown that the circRNA-miRNA-mRNA pathway may play an important role in cardiovascular diseases. For example, Zhao et al. found that the CDR1as/miR-7/CKAP4 axis is involved in the pathogenesis of abdominal aortic aneurysms (105). Li et al. confirmed that the circRNA_000203/miR-26b-5p,-140-3p/GATA4 axis aggravated cardiac hypertrophy (106). Recently, many researchers have also discovered that circRNAs have a regulatory role in the pathogenesis of AD. For example, Xu et al. found that the circ_TGFBR2/miR-29a/KLF4 axis can inhibit the progression of AD (95). In addition, various circRNAs have been found to have translational functions, and the resulting peptides also play biological roles in the occurrence and development of human diseases (107). For example, Hsa_circ_0000423 (CircPPP1R12A) encodes a peptide, circPPP1R12A-73aa, which promotes colon cancer progression by activating the Hippo-YAP signaling pathway (108). However, circRNAs translation has not been found to be related to AD formation. In conclusion, circRNAs regulate gene expression during the development of various diseases by acting as miRNA sponges, RBP sponges, and transcriptional regulators, or even directly encoding proteins (109).

### CircTGFBR2

**FIGURE 4:**
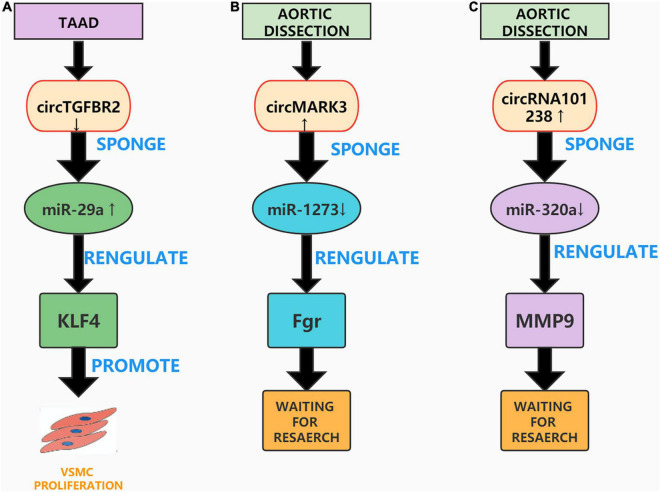
Aortic dissection associated circRNAs. **(A)** CircTGFBR2 can sponge miR-29a and affect the expression of KLF4 to promote VSMC proliferation. **(B)** The circMARK3-miR1273-Fgr pathway plays an important role in AD. **(C)** The circRNA_101238-miR-320a-MMP9 axis may also be concerned in the pathogenesis of AD.

Previous studies have found that circRNAs can be used as ceRNAs, through which sponge miRNAs compete with messenger RNAs (mRNAs) and proteins to form a complex that regulates gene expression, mRNA splicing, translation, and degradation (110). Transforming growth factor-β receptor II (TGFBR2) is a transmembrane serine threonine kinase that can be used as a tumor suppressor gene (111). For example, Li et al. confirmed that circTGFBR2 can inhibit the progression of nasopharyngeal carcinoma by sponging miR-107 and upregulating the expression of TGFBR2 (112). Studies have also shown that knockdown of the TGFBR2 gene causes aortic diseases such as Marfan syndrome and Loeys-Dietz syndrome (113). A current study by Xu et al. observed that circTGFBR2 as a ceRNA additionally performs a position in the pathogenesis of AD. They found that circTGFBR2 was downregulated in AD tissues, and performed several *in vitro* experiments to confirm that inhibiting circTGFBR2 promotes VSMCs proliferation, migration, and phenotypic conversion. Further, bioinformatic analysis confirmed that circTGFBR2 can act as an miR-29a sponge and that Krüppel-like factor 4 (KLF4) is a direct target of miR-29a in AD. Overall, the study by Xu et al. showed that circTGFBR2 can sponge miR-29a and affect the expression of KLF4 to regulate the progression of AD (114). However, this study also has limitations and lacks the establishment of animal models to verify the results of *in vitro* experiments (Figure 4A).

### CircMARK3

With the continuous improvement of high-throughput RNA sequencing (RNA-Seq) technology, many circRNAs have been discovered, however their mechanism of action in AD remains unclear (115). Recently, Tian et al. screened the circRNA expression profile of human acute Stanford A-type aortic dissection (AAAD) through RNA-Seq analysis, and constructed a circRNA-miRNA-mRNA network to explore the biological functions of circRNAs in AAAD. They speculated that the circMARK3-miR1273-Fgr pathway performs an essential position in AAAD and confirmed that the expression of circMARK3 was upregulated in the serum and aortic tissue of sufferers with AAAD. *In vitro* experiments were performed to over-express circMARK3 in primary HASMCs; these experiments printed that the expression of hsa-miR-1273g-3p was extensively reduced, and the expression of Fgr was significantly increased (116). However, Tian et al. did not further verify the circMARK3-miR1273-Fgr interaction, nor did they establish an AD animal mannequin to verify the outcomes of their *in vitro* experiments. Nonetheless, their research provides ideas and directions for future studies on the role of circMARK3 in AD (Figure 4B).

### hsa_circRNA_101238

A study found that circRNAs can be specifically expressed in tissues and developmental stages as ceRNAs and can participate in the progression of many diseases (117). Recently, Zou et al. analyzed the differential expression profile of circRNAs in AD using microarrays and constructed a circRNA-miRNA co-expression network. They found that hsa_circRNA_101238 interacted with hsa-miR-320a, hsa-miR-320b, and hsa-miR-320c and inhibited the expression of these three miRNAs. Zou et al. then analyzed the effect of hsa_circRNA_101238 as a ceRNA on the expression of MMP9. After conducting a series of *in vitro* experiments, they confirmed the low expression of hsa-miR-320a, hsa-miR-138-5p, and hsa-miR-593-5p and the excessive expression of MMP9 in TAAD tissues. Finally, they speculated that the circRNA_101238-miR-320a-MMP9 axis may be involved in the pathogenesis of AD (118). While their study is the first to propose that hsa_circRNA_101238 is concerned in the pathogenesis of AD, the research is not thorough enough. Several *in vitro* experiments and animal models are needed to confirm the effect of circRNA_101238-miR-320a-MMP9 on AD (Figure 4C).

## Summary and Perspectives

Previous studies have found that epigenetic modifications that do not change the DNA sequence play a pathogenic role in the pathogenesis of AD (97). Epigenetic modifications mainly include DNA-related histone modifications, DNA methylation, and non-coding RNA-mediated modifications (119). These modifications can regulates gene expression and protein synthesis (120). Currently, the ncRNAs in the onset of various diseases is a hot topic for research. ncRNAs interact with DNA, RNA, and proteins and become key regulators of gene expression under physiological and pathological conditions (21). This review mainly introduces the regulatory role of ncRNAs in AD.

Many studies have shown that ncRNAs (miRNAs, lncRNAs, and circRNAs) play an important role in the occurrence and development of AD; however, the underlying mechanism is not fully understood. This review describes how a variety of ncRNAs regulate the formation of AD (Table 1). Presently, the role and therapeutic potential of miRNAs (miRNA-134-5p, miRNA-145, miRNA-30a, miRNA-107, miRNA-26b) in AD is confirmed in various animal models. However, our understanding of the regulatory mechanisms of lncRNAs and circRNAs and their functions is still in its infancy (98). Notably, recent studies have found that lncRNAs regulate transcriptionally or post-transcriptionally through multiple mechanisms (99). Specifically, lncRNAs can competitively adsorb some miRNAs like natural sponges, reducing the binding of these miRNAs to their target genes, resulting in changes in the expression levels of these miRNA target genes (66). For example, lncRNA H19 regulates smooth muscle cell function through cavernous miR-193b-3p and participates in the incidence and development of AD (76). CircRNAs are a new type of endogenous non-coding RNA, which can also be used as ceRNA and miRNA sponges, thereby regulating gene expression at the post-transcriptional and transcriptional levels (24). For example, Ren et al. found that the circ_TGFBR2/miR-29a/KLF4 axis can inhibit the progression of AD (95).

There are many studies and experimental data on the regulation of ncRNAs (such as miRNAs) in AD. Data on different ncRNAs (such as lncRNAs and circRNAs) are additionally increasing rapidly. Characterization of their deregulated expression will provide possible markers for the diagnosis, prognosis, and remedy of AD. Importantly, the ceRNA regulatory network related to lncRNAs and circRNAs is now becoming a research hotspot. A higher grasp of the molecular mechanism of ncRNA-mediated AD may additionally assist decide appropriate intervention targets to control the formation or development of AD. However, the existing studies have some limitations. First, research on the regulatory role of ncRNAs in AD focuses on the effect of miRNAs or lncRNAs on VSMCs. Subsequent research needs to be extended to include other cellular components of blood vessels, such as ECs, fibroblasts, and macrophages. In addition, the research on the molecular mechanism of AD pathogenesis requires further evidence because many researchers have only completed cell function experiments and have not established animal models for verification. Finally, in addition to miRNAs, lncRNAs, and circRNAs, other ncRNAs, such as siRNAs and piRNAs may also be involved in the development of cardiovascular disease, and their function needs to be evaluated.

## Author Contributions

Y-YH collected relevant literature data and wrote the manuscript. X-MC provided help and advice. X-NW designed and directed the study. All authors contributed to the article and approved the submitted version.

## Conflict of Interest

The authors declare that the research was conducted in the absence of any commercial or financial relationships that could be construed as a potential conflict of interest.

## Publisher’s Note

All claims expressed in this article are solely those of the authors and do not necessarily represent those of their affiliated organizations, or those of the publisher, the editors and the reviewers. Any product that may be evaluated in this article, or claim that may be made by its manufacturer, is not guaranteed or endorsed by the publisher.
